# New Insights into the (A)Synchronicity of Diels–Alder Reactions: A Theoretical Study Based on the Reaction Force Analysis and Atomic Resolution of Energy Derivatives

**DOI:** 10.3390/molecules27051546

**Published:** 2022-02-25

**Authors:** Bienfait Kabuyaya Isamura, Kevin Alan Lobb

**Affiliations:** 1 Department of Chemistry, Rhodes University, Makhanda 6140, South Africa; isamurabft@gmail.com; 2 Research Unit in BioInformatics (RUBi), Rhodes University, Makhanda 6140, South Africa

**Keywords:** Diels–Alder reaction, AMADAR, reaction force analysis, natural population analysis, Hellman–Feynman forces

## Abstract

In the present manuscript, we report new insights into the concept of (a)synchronicity in Diels–Alder (DA) reactions in the framework of the reaction force analysis in conjunction with natural population calculations and the atomic resolution of energy derivatives along the intrinsic reaction coordinate (IRC) path. Our findings suggest that the DA reaction transitions from a preferentially concerted mechanism to a stepwise one in a 0.10 Å window of synchronicity indices ranging from 0.90 to 1.00 Å. We have also shown that the relative position of the global minimum of the reaction force constant with respect to the TS is an alternative and quantifiable indicator of the (a)synchronicity in DA reactions. Moreover, the atomic resolution of energy derivatives reveals that the mechanism of the DA reaction involves two inner elementary processes associated with the formation of each of the two C-C bonds. This resolution goes on to indicate that, in asynchronous reactions, the driving and retarding components of the reaction force are mostly due to the fast and slow-forming C-C bonds (elementary processes) respectively, while in synchronous reactions, both elementary processes retard and drive the process concomitantly and equivalently.

## 1. Introduction

The [4 + 2] cycloaddition reaction between a conjugated diene and a substituted alkene (dienophile) is known as the Diels–Alder (DA) reaction [1]. This chemical transformation is one of the most commonly encountered reactions in organic chemistry, allowing for the formation of six-membered rings with up to four stereogenic centers [2]. Since its discovery in 1928 by Otto Diels and Kurt Alder [3], the DA reaction has found many applications, especially in the total synthesis of natural products [4,5,6]. Some of the most striking examples include the synthesis of the steroids cortisone and cholesterol by Woodward et al. [7] and the preparation of disodium prephenate, a biosynthetic precursor of the amino acids phenylalanine and tyrosine [8]. In addition to its relevance in natural products chemistry, the DA reaction and its variants are also utilized for many other purposes, such as in the design of polymers and drug delivery systems [9,10,11], as well as in the synthesis of agrochemicals [12,13].

Over the past decades, the mechanism of the Diels–Alder (DA) reaction has been the subject of numerous experimental [14] and theoretical [15] studies. Despite a handful of works mentioning DA reactions following a stepwise mechanism [16], the debate around the mechanistic route of DA reactions has ended up suggesting that most DA reactions proceed through a concerted pathway [17]. However, being concerted does not imply that all [4 + 2] cycloadditions are synchronous because, on a time-resolved scale, there is no guarantee that the two nascent C-C bonds will be formed concomitantly. According to Dewar [18], a synchronous multi-bond reaction is one in which the bonds are formed in unison. Nendel et al. reported that asynchronous DA reactions tend to follow a stepwise mechanism and vice-versa [19]. From a computational view, the simplest and most used indicator of the DA degree of synchronicity measures the difference between the lengths of the two emerging C-C bonds at the TS. Several other definitions have been proposed to estimate the synchronicity of cycloaddition reactions, such as considering the relative variation of Wiberg indices of all the bonds directly involved in the active site of the reaction [20,21]. Merino et al. also showed that topological analyses could provide information regarding synchronicity that, often, is not reflected in the geometry of transition state structures [22]. However, the former structural estimator is still the most used.

For a long time, the widespread opinion has been that unsymmetrically substituted dienes/dienophiles lead to unsymmetrical TS and thus asynchronous mechanisms [19]. However, Souza et al. found that this was not correct on a time-resolved scale and provided strong evidence showing that even unsymmetrical antagonists could proceed via synchronous routes [23]. Such controversies provide the rationale to dig deeper into the essence of (a)synchronicity in DA reactions. In this vein, various studies have attempted to elucidate the origin of the (a)synchronicity in DA reactions using different approaches, including, but not restricted to, the frontier molecular orbital theory [24], molecular electron density theory [25] and activation strain model [26]. In particular, the reaction force analysis has revealed that the second derivative of the system’s energy with respect to the reaction coordinate (known as the reaction force constant) is a good indicator of the synchronicity of DA reactions [27].

While all of these studies have significantly contributed to the knowledge surrounding the (a)synchronicity of DA reactions, there are, nevertheless, a variety of pertinent questions and omissions regarding this that still need to be addressed. First, it is to be noted that many previous studies have investigated specific or small datasets often made of very similar DA reactions. Obviously, this raises concerns about the extrapolation of their findings. Second, to the best of our knowledge, no one has tried to gain a thorough understanding of the individual role(s) of each atom or group of atoms, especially those involved at the reactive site, in the overall (a)synchronicity of DA reactions. Unfortunately, this fundamental question cannot be answered even in the context of the standard reaction force analysis (RFA), which only provides an explanation of the mechanism in terms of global energy derivatives [28].

Assessing the individual role of specific atoms or fragments in the (a)synchronicity of DA reactions in the framework of the RFA supposes that energy derivatives along the reaction path, namely the reaction force F and reaction force constant κ, have been decomposed into atomic contributions. In 2016, Ledrzejewski, Ordon and Komorowski (LOK) [29] demonstrated the feasibility of doing such a decomposition by referring to the Hellman–Feynman theorem, which defines the nature of individual forces acting on nuclei in molecular systems [30]. Their promising approach goes beyond the standard reaction force analysis, bringing up the missing breaks for an accurate atomic resolution of the reactive force F and reaction force constant κ. We have recently reported a python-based package named AMADAR, which implements their formalism as well as the standard reaction force analysis in two special modules [31].

In the present study, the reaction force analysis is used in conjunction with natural population calculations and the LOK atomic resolution of F and κ to get insight into the (a)synchronicity of DA reactions. This study draws its particularity from the large size of the dataset used and the fact that it is probably the first work reporting the application of the LOK formalism on DA reactions. It has been found that the mechanism of DA reactions involves two elementary processes whose interplay defines the nature and magnitude of the reaction force and reaction force constant. The Wiberg bond order analysis is shown to provide an insightful picture of the process of bond formation/breaking along the reaction path. Moreover, our results confirm some previous findings while adjusting or complementing others.

## 2. Results and Discussion

### 2.1. The Dataset

The AMADAR pipeline was fed with 2000 DA cycloadducts extracted from the ZINC database. About 1910 were successfully converted back into the corresponding TSs, representing a success rate of 95%. Each of these structures was confirmed to possess a unique imaginary frequency in a range between 400 and 800 i. The optimized geometries and the numbering used in this study to denote all the predicted TSs and corresponding reactions are provided in the Supplementary Materials. The two main causes of errors were basis set inconsistencies and failures in geometry convergence. As expected, basis set errors were returned for all the systems containing iodine atoms, which cannot be described by the valence-split double zeta 6-31G(d) basis set. Errors in convergence criteria were observed for very large systems, for which the maximum number of iteration steps was exceeded before reaching the minimum and resulted in the abortion of the calculations. A separate module is being developed to address these issues, and some minor ones, in a systematic way and will be integrated into the next release of the package.

#### 2.1.1. Heterogeneity

**Figure 1:**
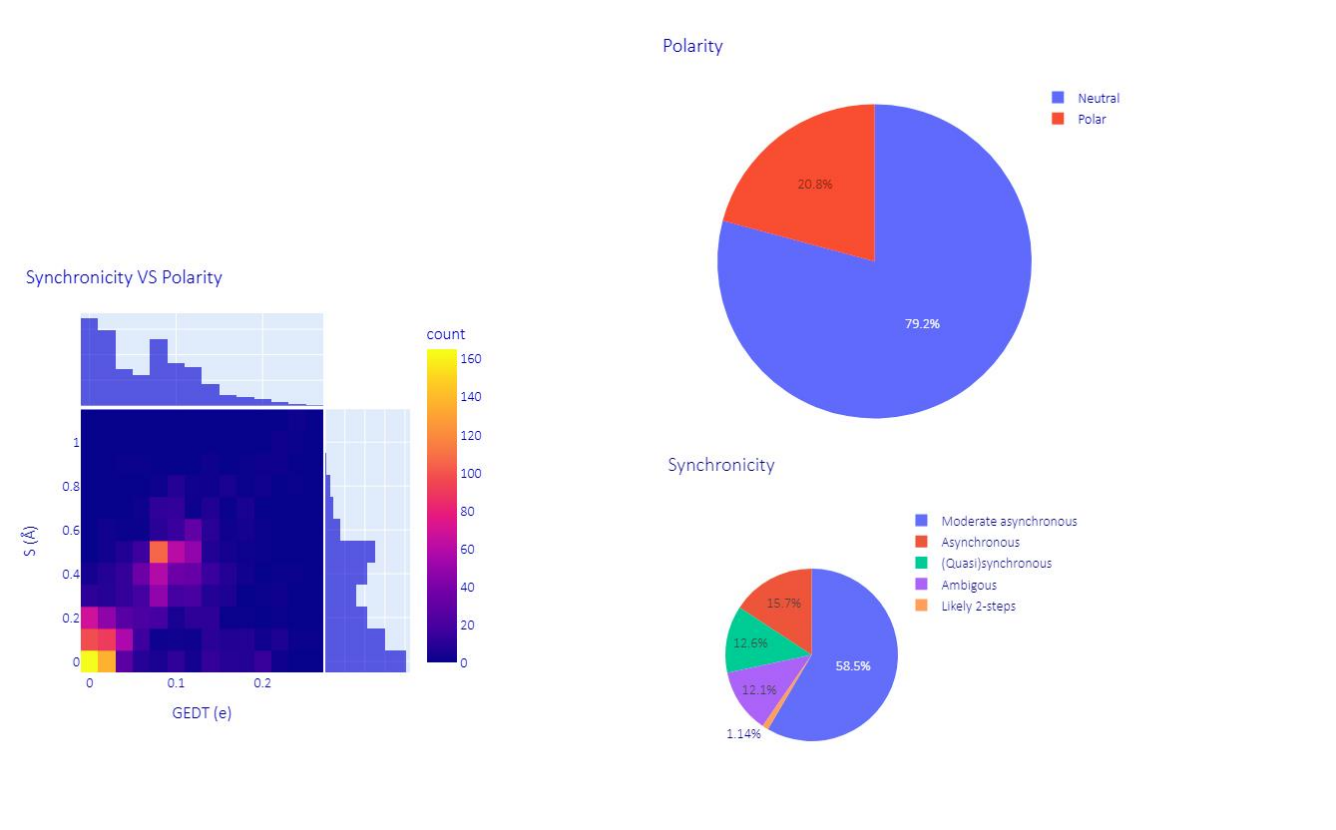
Overview of the dataset: synchronicity and polarity of the reactions.

To assess the heterogeneity of the dataset, all 2000 cycloadducts were retro-converted into their initial diene and dienophiles, making use of some functionalities of the AMADAR program. This led to about 90 and 1140 different dienes and dienophiles, respectively. Here, 1,3-butadiene was the most frequent diene, involved in ~55% of the DA reactions, followed by 1,3-cyclopentadiene and 1,3-cyclohexadiene, which were associated with 10% of the reactions. It must be noted that all the remaining dienes were substitutional derivatives of the previous. The ethylene molecule was the most popular dienophile, representing about 30% of the entire population. The comprehensive list of reactants and cycloadduct SMILES strings is provided in a separate file as part of the Supplementary Materials. The dataset of TSs generated covers a wide range of electronic and structural features. Its diversity was examined in terms of polarity and synchronicity (Figure 1).

#### 2.1.2. Polarity

The polarity of each DA reaction was estimated by measuring the magnitude of the electron density exchanged between the diene and dienophile at the TS. This amount is known as the “global electron density transfer” (GEDT) in the framework of molecular electron density theory [32]. Predicted GEDT values ranged between 0.00 and 0.26 e. According to the classification of DA reactions proposed here [33] based on GEDT values, our dataset encompasses 79.2% of neutral DA reactions (0 < GEDT < 0.15 e) and 20.8% of polar ones (0 0.15 e < GEDT < 0.45 e). Next to these types, ionic DA reactions are also known in the literature, in which at least one of the two reactants is charged, resulting in GEDT values greater or equal to 0.45 e [34]. Ionic DA reactions are found in the synthetic scheme of many multistep reactions, such as in the synthesis of chromans through the oxo-Povarov reaction, where the initial step is an ionic DA between cationic aryl oxonium and an alkene [35].

While examining the direction of the charge transfer measured at the TSs, we noticed that the dienes (positively charged) had released their electron density onto the dienophiles in 73% of the predicted TSs. This observation suggests that most of the systems investigated had a normal electron demand or normal flux. Moreover, in opposition to Domingo and his coworkers, who reported a good correlation between the polarity and the activation energy of DA reactions (R^2^ = 0.99) [29], we did not observe such a significant correlation in our dataset (R^2^ = 0.32, Figure S1, Supplementary Materials). This discrepancy can be first ascribed to the high heterogeneity of our dataset as compared to theirs, which was formed of similar DA reactions involving a unique diene (1,3-cyclopentadiene) against twelve substituted ethylenes. Another reason may be the fact that their study involved only polar DA reactions, while our dataset was mostly made of neutral DA processes. Therefore, it may be postulated that high correlations between GEDT values and activation energies should only be expected within groups of very similar and polar DA reactions.

#### 2.1.3. (A)Synchronicity

The (a)synchronicity of the predicted TSs (DA reactions) was assessed using the widely admitted structural index S, defined as the difference between the lengths of the two emerging C-C bonds at the TS. As shown in Figure 1, 58.5% of the reactions considered in this study are moderate asynchronous processes, with S values between 0.25 Å and 0.55 Å. The rest of systems comprise 12.6% of (quasi)synchronous (0.00 < S < 0.20 Å), 15.7% of asynchronous (0.60 < S < 0.90 Å) and 1.1% of likely two-step DA reactions (S > 0.95Å). This alternative classification of DA reactions is proposed here based on the degree of (a)synchronicity. It is worth noting that DA reactions in each of these four subgroups share the same patterns on the profile of the reaction force constant along the IRC path (Section 2.2.2), which suggests that they have similar mechanistic features regardless of their polarity and electron density flux, and can be grouped.

**Figure 2:**
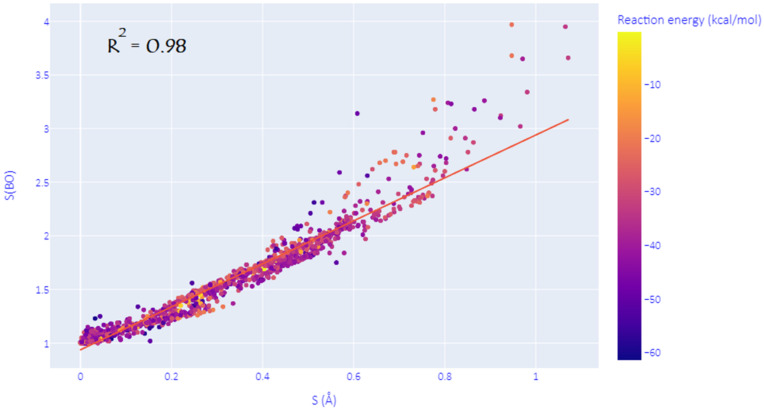
Correlation between the structural and electronic synchronicity indices.

As mentioned in the introduction, the structural synchronicity index S is not a unique way to assess the degree of synchronicity in DA reactions, and many other viable descriptors may be used instead. This hypothesis is confirmed here by investigating the Wiberg bond orders (WBO) of the two nascent C-C bonds at all the predicted TSs. Indeed, as shown in Figure 2, we have found that an electronic equivalent of S, noted S(BO), can be defined as the natural logarithm of the ratio (>1) of WBOs of the two emerging C-C bonds at the TS. The correlation coefficient of R^2^ = 0.98 between S and S(BO) proves that these descriptors can be used interchangeably to measure the (a)synchronicity of DA reactions. However, it has to be noted that this correlation is more marked for (quasi)synchronous and moderate asynchronous processes and tends to deviate as the reaction becomes more and more asynchronous. In the discussion below, we have chosen to consider the commonly used index S for consistency with previous studies.

### 2.2. Reaction Force Analysis

**Figure 3:**
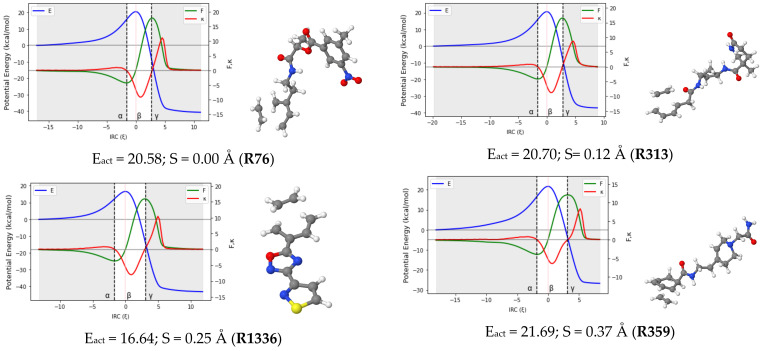

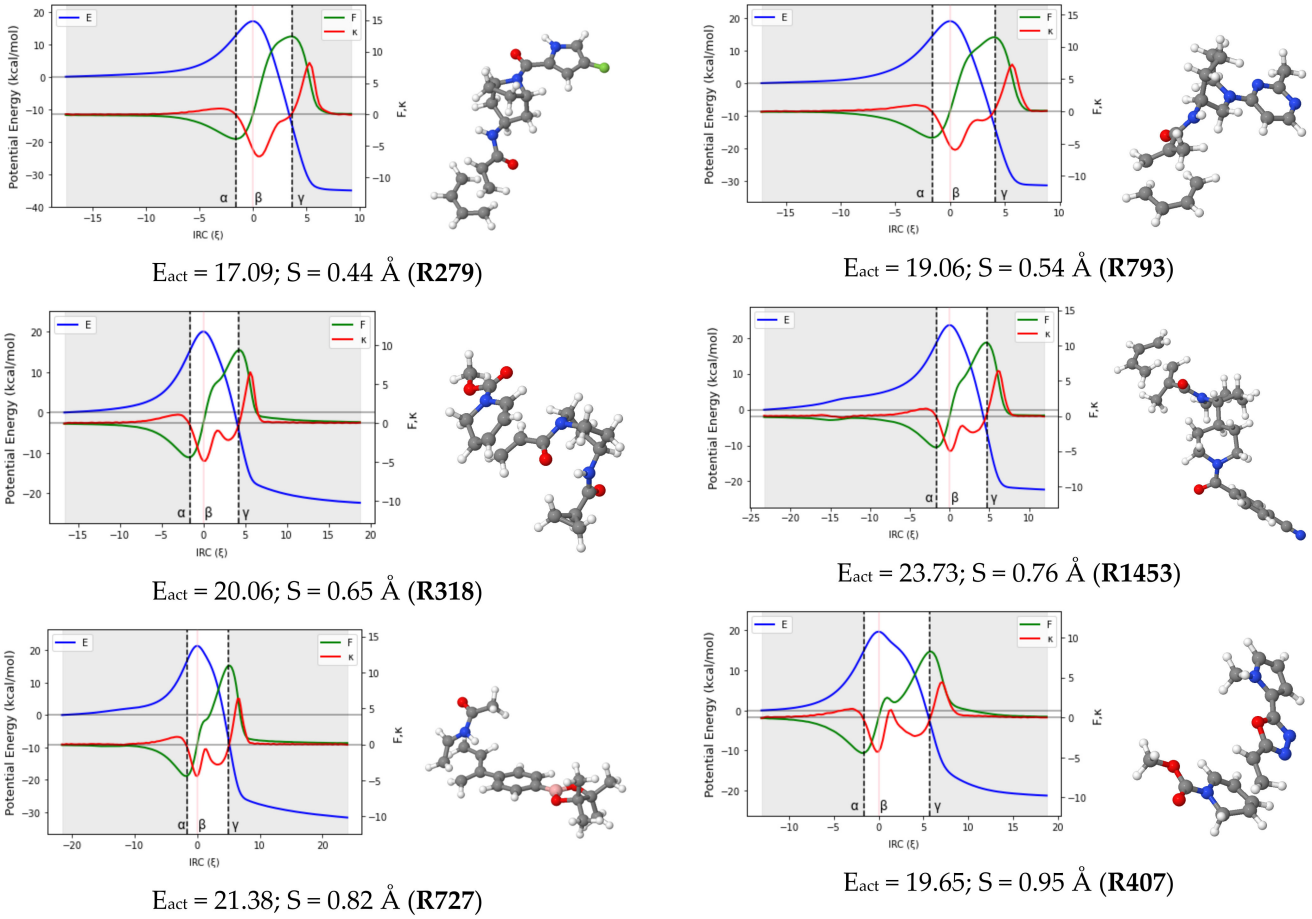
RFA plots for a representative set of ten DA reactions. TSs structures, activation energy and synchronicity of the corresponding reactions are also given. Each reaction is identified by its ID in line with the numb.

Reaction force analysis (RFA) was performed for 150 Diels–Alder reactions with different structural and electronic features. Figure 3 presents the profiles of the potential energy E, the reaction force F, and the reaction force constant κ for a representative set of ten reactions systematically selected to cover a wide range of synchronicity indices. Be advised that qualitatively equivalent conclusions to those reported below were inferred from any random set of reactions with similar degrees of synchronicity. The position of the classical TS is indicated by β, while the minimum and maximum of the reaction force F are denoted by the Greek letters α and γ, respectively. Table 1 collects the two components of the activation energy, E_act,1_ and E_act,2_, as well as the polarity and synchronicity of the corresponding DA reactions for an illustrative set of twenty systems.

#### 2.2.1. Potential Energy

It is clear from Figure 3 that the profile of the potential energy is insensitive to the synchronicity of DA reactions. Only strongly asynchronous DA reactions (S > 0.90 Å) present a barely noticeable inflection point in the PE curve in the second section of the TS region. As such, the potential energy curve cannot serve as an indicator for the synchronicity of DA reactions. 

Moreover, as shown in Table 1, E_act,1_ and E_act,2_ values suggest that the most important amount of the activation energy is consumed in the preparation phase, taking, on average, roughly 70–75% of the energy required to bring the system up to its transition state. For example, the E_act_ of the (quasi) synchronous **R73** DA reaction (20.6 kcal/mol) is composed of 15.7 kcal/mol and 4.8 kcal/mol, involved, respectively, in the preparation phase and the first part of the TS region at the B3LYP/6-31G(d) level. Similarly, the E_act_ of the asynchronous **R1453** DA reaction is predicted to be 23.8 kcal/mol, with E_act,1_ and E_act,2_ accounting for 18.9 and 4.9 kcal/mol, respectively. This rule applies to all the reactions investigated regardless of their polarity and synchronicity and agrees with the study by Mancera et al., who investigated the DA reactions between a 9,10-disubstituted chiral anthracene and a short series of dienophiles, and found that more than 70% of the activation energy was involved in the preparation phase [36]. While this may appear to be a common feature for all DA reactions, it should not be generalized to other reactions. For example, an exception is the SN_2_ reaction between CH_3_Cl and H_2_O. This reaction requires approximately the same amount of energy during the preparation step (R →α) and the first part of the TS region, estimated at +27.0 and +25.0 kcal/mol, respectively, at the B3LYP/6-31G(d) level [37].

#### 2.2.2. Reaction Force and Reaction Force Constant Profiles

Figure 3 reveals that the reaction force F switches from negative (retarding) to positive (driving) values when the system crosses the classical TS. This observation indicates first the existence of a dominating retarding force in the preparation phase that opposes structural changes in the system [28]. As the system approaches the TS region, a positive driving force arises and steadily foils the retarding one. The two forces acting on the system keep interplaying up to the TS, where their magnitudes exactly counterbalance. As such, the TS should be regarded as a mechanical equilibrium point on the IRC path, as it denotes the moment when the resultant of all forces acting on the system is null. After the TS, the driving force wins over the retarding force and drags the system to the product state.

In the framework of the activation strain model (ASM) developed by Bickelhaupt and coworkers [38], the potential energy E at each point of the IRC path is decomposed into the strain and interaction components. By combining the ASM and the RFA, Politzer et al. were able to quantify the retarding and driving forces that compose the total reaction force of DA reactions [39]. Their study showed that the retarding force observed in the preparation phase is the sum of positive strain and repulsive forces. The two components were identified with the opposite gradient of the strain and interaction energy constituents of a system’s potential energy. In Section 2.4.1, we provide another picture of the same phenomenon, where the reaction force appears to be composed of two components associated with the formation of each C-C bond. The contribution of each component to the reaction force depends on the degree of (a)synchronicity of the reaction.

The profile of the reaction force constant is the most affected by the change in the reaction’s (a)synchronicity. Particularly, it reveals some interesting patterns that depend on the degree of (a)synchronicity of DA reactions. More than the reaction force, the reaction force constant appears to be a good indicator of the (a)synchronicity in DA reactions. Yepes et al. were the first to report this observation in their investigation of a series of seven DA reactions involving 1,3-cyclopentadiene and cyanoethylenes [27].

Regarding the patterns arising from the profile of κ in Figure 3, one can categorize DA reactions into four subsets, including (quasi)synchronous (0.00 < S (Å) <0.20), moderate asynchronous (0.25 < S (Å) <0.55), fully asynchronous (0.60 < S (Å) <0.90) and likely two-step (S (Å) ≥ 0.95) processes. Note that these boundaries are not continuous. This is because the DA reactions within the gaps may be of either type depending on the structural and electronic peculiarities of each reaction. However, in every subgroup, some common features emerge and are as follows: in (quasi-)synchronous DA reactions, the reaction force constant κ has a unique minimum in the TS region. At the same time, F increases continuously in the same region before crossing a sharp maximum. As the reaction becomes more and more asynchronous, some striking changes are observed on the graph of F and κ. For moderate asynchronicity (0.25 < S (Å) < 0.55), κ presents not only a minimum in the TS region but also an inflection point (shoulder) located after the minimum in the direction of the reaction coordinate. As we shall show in the next section, this sudden appearance of an inflection point in the TS region of κ can be associated with the formation of the first C-C bonds. A slight change is also observed in the graph of F as it flattens around its maximum. For asynchronous DA reactions (0.60 < S (Å) < 0.90), the reaction force F shows an inflection point in the TS region, while the reaction force constant κ presents two minima and a maximum (between these minima) in the same region. These characteristics have been observed in a smaller dataset of other DA reactions [27,36].

Let it be highlighted here that the sign of the reaction force constant over the TS region can be very informative. In the case of fully synchronous to asynchronous DA reactions, it remains negative all along the TS region. However, when the reaction becomes very asynchronous (S > 0.95 Å), the maximum of κ in the TS region is lifted to more and more positive values, and a new inflection point is (barely) noticeable in the TS region of E. A very drastic change is also observed in the profile of the reaction force: the characteristic inflection point observed in the TS region of asynchronous reactions falls apart into a new local minimum and a local maximum. Similar observations were reported by Politzer et al. [40] for a series of double proton transfer reactions. To the best of our knowledge, this is the first time this observation has been reported in the case of DA reactions.

From a mechanistic view, the presence of a positive local maximum in the TS region on the profile of κ has been associated with the emergence of a metastable intermediate in the region situated between the new local extrema (maximum and minimum) of the reaction force [40]. To check this hypothesis, vibrational frequency calculations of all the geometries located in the second part of the TS region for the reactions **R313** (S = 0.12 Å), **R279** (S = 0.44 Å), **R727** (S = 0.82 Å), and **R404** (S = 0.95 Å) were carried out. The results obtained revealed the existence of one structure with a unique imaginary frequency (a TS) in the TS region of **R404** and any in those of **R313, R279,** and **R727**. This finding suggests a more complex mechanistic pathway for **R404** and may support the assumption that DA reactions with a synchronicity index higher than 0.95 Å may follow a stepwise route. However, due to the peculiarity of each system, the situation cannot be generalized. It is then more cautious to declare that the transition between a preferentially concerted mechanism and a two-step pathway may be located somewhere between S = 0.90 Å and S = 1.00 Å. Therefore, very asynchronous DA reactions (S = 0.95 Å and S = 1.00 Å) are likely to proceed through a two-step non-concerted pathway, with a diradical or zwitterionic intermediate. Although more evidence is still needed to confirm this, this temporary conclusion is consistent with previous findings [41], and more interestingly, it adds a relevant detail regarding the range of synchronicity indexes where the transition from the concerted to the stepwise routes may be observed.

### 2.3. Natural Population Analysis

**Figure 4:**
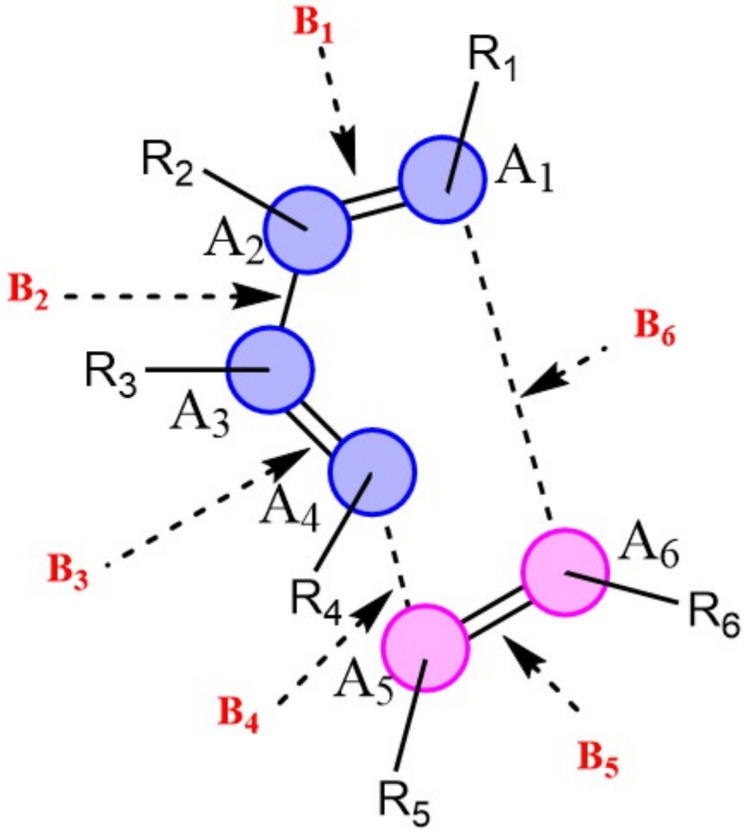
Reactive site of DA reactions. Atoms of the dienophile and diene are indicated in blue and pink, respectively.

**Figure 5:**
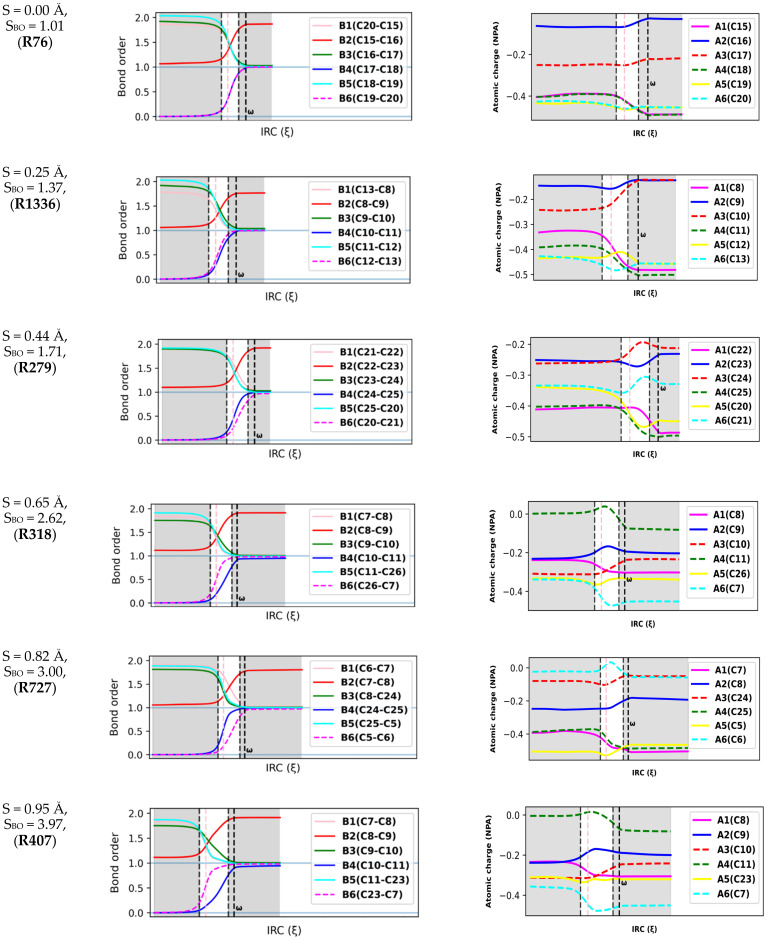
Bond order and atomic charge analysis of an illustrative set of six Diels–Alder reactions. The position of the maximum of κ is marked (ω). The TS regions are shown in white, and the TS is shown by the pink dashed vertical lines.

Natural population calculations were performed on the successive configurations along the IRC paths of 150 DA reacting systems to assess the bond breaking/forming processes during these reactions. Figure 4 depicts the convention used herein to designate the six atoms (A1-A6) and six bonds (B1-B6) involved in the reactive site of DA reactions. In Figure 5, the Wiberg bond orders of B1-B6 as well as atomic charges on A1-A6 are plotted against the reaction coordinate for a representative set of six DA reactions (0 < S(Å) < 1). These reactions cover a wide range of synchronicity indices. Similar plots for five other DA reactions are provided in Supplementary Materials, Figure S2). The position of the global maximum of the reaction force constant, noted ω, is also indicated.

Figure 5 reveals that bond orders and atomic charges remain almost constant over the preparation phase. This finding suggests that electronic effects are not pronounced during this step and confirms previous findings by Burda et al. [42]. Furthermore, the constancy of atomic charges in the reactive site during the preparation phase reveals that the overall interaction between the dienes and dienophiles is mostly of an electrostatic nature since very few charge transfers are experienced between the antagonists. This agrees well with Politzer et al., who found that the diene and dienophile experienced mostly a purely repulsive electrostatic interaction in the preparation phase due to the interaction between their π clouds [39]. 

The six bonds in the reactive site begin to change considerably before entering the TS region. Moreover, in agreement with the fact that the TS region is admitted to be dominated by electronic changes [43], the most important changes in electronic properties (bond orders and atomic charges) take place in the TS region. A striking feature from Figure 5 is that the second (global) maximum of κ (ω) marks the point where all the bonds and atomic charges in the reactive site reach their asymptotic values and stop changing. At that point, all the bonds, and especially the second C-C bond, can then be considered to be completely formed. Therefore, we can postulate that ω represents the moment of the reaction when DA reacting systems become topological equivalents of the cycloadducts, as they now contain the same types and number of bonds as the cycloadducts.

The relative positions of the B4 and B6 curves constitute another indicator of the synchronicity of DA reactions. For a fully synchronous DA reaction, the two curves are perfectly overlaid, suggesting the two bonds form at the same rate and time. At the position of the classical TS, the nascent C-C bonds are almost halfway in the process of their formation, with bond orders close to 0.5. Therefore, one can assume that synchronous DA reactions proceed through cyclic TSs where the terminal C atoms are non-covalently bound. For asynchronous processes, one of the two C-C bonds is delayed, and this deviation is as much pronounced as the reaction is asynchronous. In addition, the first C-C bond is (almost) completely formed in the TS region, reaching a bond order >0.95. In very asynchronous processes such as **R404** and **R727**, Figure 5 does not illustrate clearly whether the TSs (vertical line in pink) should be considered as cyclic or not since the bond order of the slow-forming bond is still very small at the classical TS (<0.2). 

However, a quick topological QTAIM analysis of the TS of **R404** and **R727** revealed the existence of critical bond points between the two pairs of terminal C atoms, which does not support the assumption of an acyclic structure. Similar findings were obtained by Hammoudan et al. on a set of 3 asynchronous hetero-DA reactions based on the QTAIM approach [44]. Therefore, based on the results we have thus far, there is insufficient proof to deny the cyclicality of asynchronous DA TSs, at least for the systems we have investigated.

Figure 5 reveals that both bond orders and atomic charges remain almost invariant over the preparation and relaxation phases. This finding suggests that electronic effects are not very pronounced during these steps and confirms previous findings by Burda et al. [42]. Furthermore, the constancy of atomic charges in the reactive site during the preparation phase may indicate that the overall interactions between the dienes and dienophiles are mostly of an electrostatic nature since only tiny electron densities are transferred between the antagonists. This agrees well with Politzer et al., who found that the diene and dienophile mostly experienced a purely repulsive electrostatic interaction in the preparation phase due to the interaction between their π clouds [39]. The six bonds in the reactive site begin to change considerably before entering the TS region. The most important changes in bond orders take place in the TS region, in agreement with the fact that this region is admitted to be dominated by electronic changes [43]. A striking feature in Figure 5 is that the second (global) maximum of κ (noted ω) marks the point where all the bonds and atomic charges in the reactive site stop changing as they have reached their asymptotic values. At that point, all the bonds, and especially the second C-C bond, can then be considered to be completely formed. Therefore, we can postulate that ω represents the moment of the reaction when DA reacting systems become topological equivalents of the cycloadducts, as they now contain the same types and number of bonds as the cycloadducts.

The relative positions of the B4 and B6 curves constitute another indicator of the synchronicity of DA reactions. For a fully synchronous DA reaction, the two curves are perfectly overlaid, suggesting the two bonds form at the same rate and time. At the position of the classical TS, the nascent C-C bonds are almost halfway in the process of their formation, with bond orders around 0.5. Therefore, one can assume that synchronous DA reactions proceed through cyclic TSs where the terminal C atoms are non-covalently bound. For asynchronous processes, one of the two C-C bonds is delayed, and this deviation is as much pronounced as the reaction is asynchronous. In addition, the first C-C bond is (almost) completely formed in the TS region, reaching a bond order >0.95. In very asynchronous processes such as **R404** and **R727**, Figure 5 does not clearly address whether the TSs should be considered as cyclic or not since the bond order of the slow-forming bond is still very small at the classical TS (<0.25). However, a quick topological QTAIM analysis of the TS of **R404** and **R727** revealed the existence of critical bond points between the two pairs of terminal C atoms, which gained the assumption of an acyclic structure. Similar findings were obtained by Hammoudan et al. on a set of 3 asynchronous hetero-DA reactions based on the QTAIM approach [44]. Therefore, based on the results obtained thus far, there is insufficient proof to deny the cyclicality of asynchronous DA TSs, at least for the systems we have investigated.

The process of bond breaking/forming during DA reactions has also been investigated using other tools in previous studies. The bond evolution theory (BET) has probably been one of the most promising approaches for this purpose. It is a combination of the ELF topological analysis [45] and the catastrophe theory [46]. Berski et al. applied the BET to the normal-electron demand DA reaction between 1,3-butadiene (BD) and acrolein (Acr), and the inverse electron-demand one between 2,4-pentadienal and methyl vinyl ether. These authors found that the entire process could be split into 11 or 10 catastrophe sequences, respectively, including the cusp and fold types of catastrophes. Cusp catastrophes were associated with the reduction of the three C=C bonds into C-C bonds, as well as with the formation of the two new C-C bonds. Fold catastrophes resulted from the concentration of the electron density on terminal C atoms, which preceded the formation of new C-C bonds [47]. Moreover, using the ELF topological analysis, Domingo et al. demonstrated that the formation of the new C-C bonds in polar DA reactions was induced by the merging of two monosynaptic basins (pseudo-radical) located on terminal C atoms into a unique bisynaptic basin [25].

### 2.4. Atomic Resolution of Energy Derivatives

**Figure 6:**
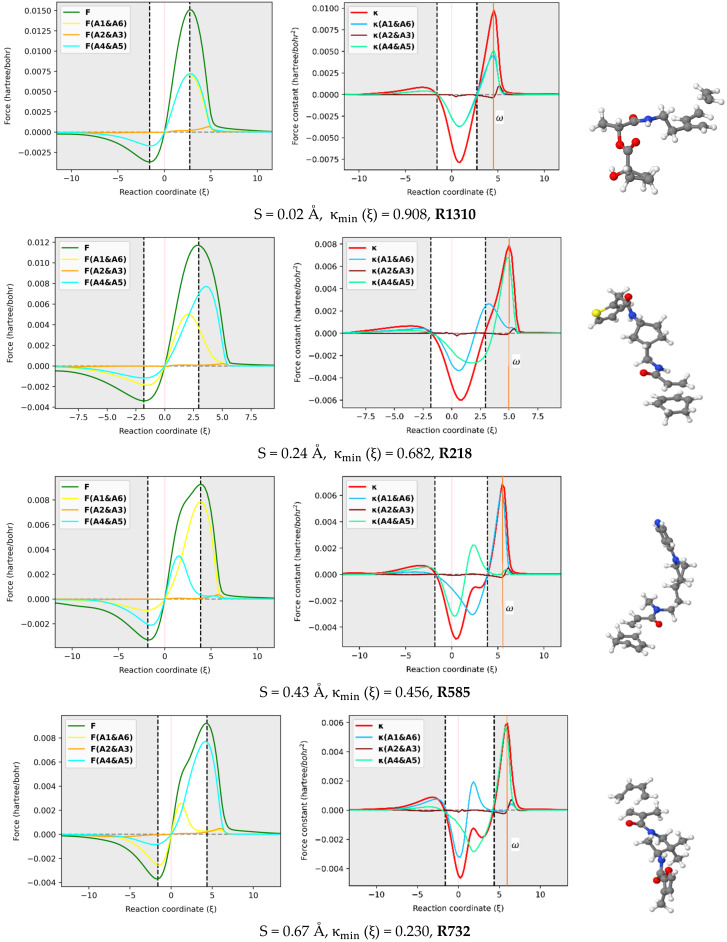

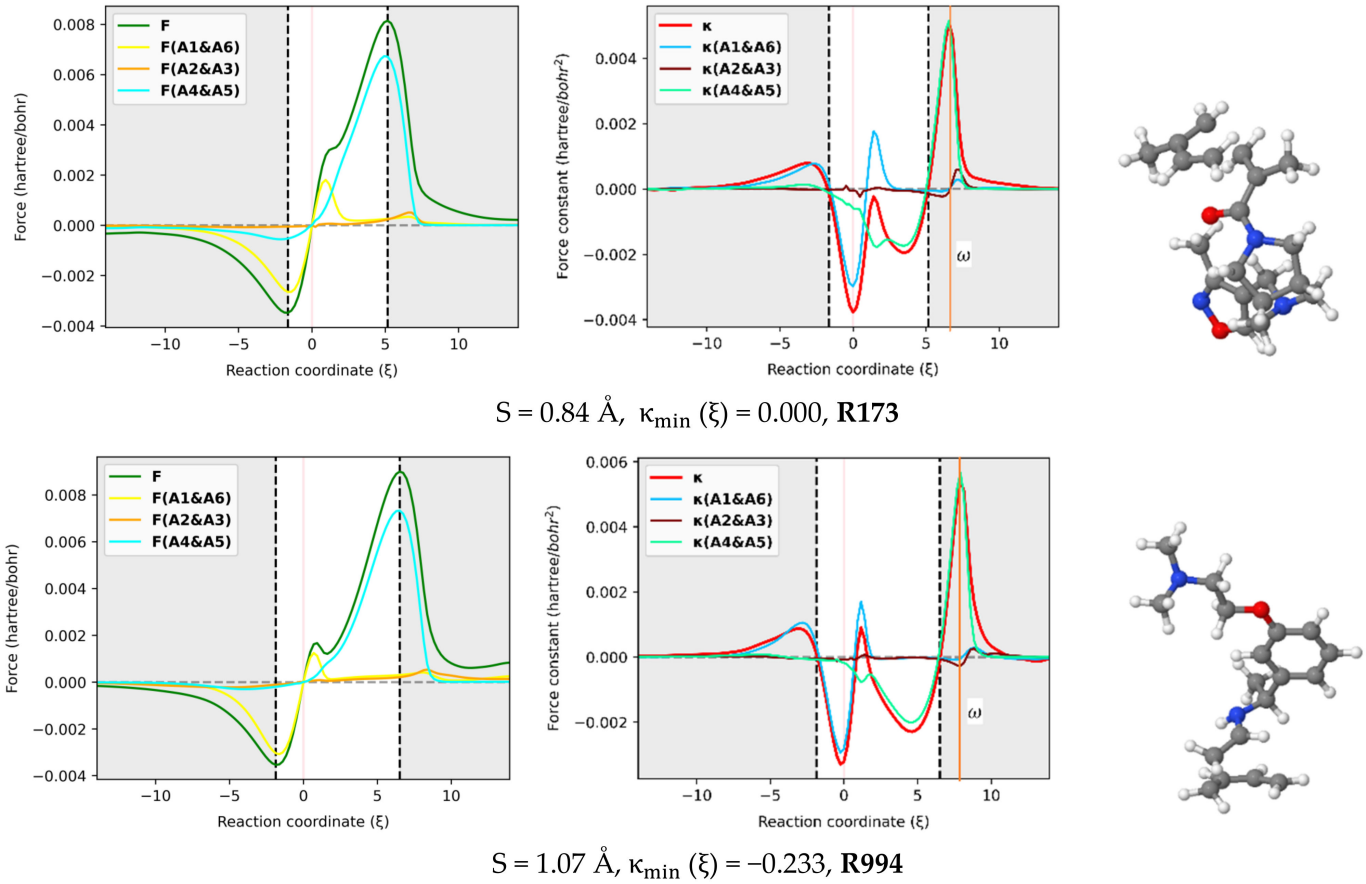
In-pair atomic resolution of the reaction force F and reaction force constant κ for atoms in the reactive site of a representative set of six DA reactions with synchronicity indexes between 0.02 Å to S = 1.07 Å.

The reaction force F and reaction force constant κ were decomposed into three pairs of contributions (A1 & A6, A2 & A3, A4 & A5) involving the six atoms in the reactive site using the approach proposed here [29] and implemented in our AMADAR program. Figure 6 depicts these contributions for an illustrated set of six DA reactions covering a range of synchronicity indices from 0.02 to 1.02 Å. It should be noted that qualitatively equivalent conclusions to those reported below were deduced by picking up any random set of other DA reactions.

#### 2.4.1. Reaction Force Decomposition

The atomic resolution of the reaction force F was first examined. Figure 6 shows that the terminal C atoms (A1 & A6 and A4 & A5) bring the most important coupled contributions to F, while those originating from the two others (A2 & A3) are almost negligible. This observation can be rationalized by the fact that A1, A4, A5, and A6 are directly involved in the interaction between the diene and dienophile. As such, they must experience the greatest electronic effects as the reaction goes through, which results in considerable electrostatic interactions and Hellman–Feynman forces [48]. Note that the atomic contribution to the reaction force, noted F_A_ (ξ) for a given atom A, is obtained as the scalar product of the Hellman–Feynman force F_A_ acting on the corresponding nucleus and the vector denoting the change of the nuclear position $\frac{\partial R_{A}}{\partial \xi }$. Since all the atoms in the reactive site move considerably during the process, the only explanation for the insignificant contributions from A2 and A3 seems to be the existence of small Hellman–Feynman forces acting on their respective nuclei.

The most striking feature from Figure 6 is that the overall profile of the reaction force F is dictated by two dominant contributions stemming from the two pairs of interacting atoms. This finding suggests that the mechanism of the DA reaction can be understood in terms of two inner elementary processes identified with the formation of the two C-C bonds. Each of the two processes has its appropriate critical points, and it is only their combination that gives rise to the observed profile. One can follow these individual critical points to get insight into the formation of the two C-C bonds. In this view, it comes out of Figure 6 that both processes have their global minima whose position on the IRC path matches that of the reaction force F, with a relative magnitude that depends on the degree of synchronicity of the DA reaction. For synchronous reactions, the global minimum and maximum of two curves are situated at about halfway of the curves of the total reaction force, let alone the fact that the curves superimpose over the IRC path. This suggests that the two nascent C-C bonds equally oppose and drive the structural change taking place in the reacting system throughout the process. As the DA reaction becomes more asynchronous, the main retarding force comes from the fast-forming bond, while the second bond brings the most driving force towards the formation of the product. Therefore, one mechanistic difference between synchronous and asynchronous DA reactions seems to be that, in asynchronous reactions, the driving and retarding forces are mainly caused by two separate elementary processes, while in the case of synchronous reactions, both elementary processes retard and drive the process concomitantly and equivalently. Nevertheless, it must be noted that, for asynchronous DA reactions, the steep increase of F in the surrounding of the classical TS is still due to the fast-forming bond, whose positive effect is more pronounced in that area than that of the second bond, before decaying quickly and thereby leaving the control to the second bond that drives the process just after.

#### 2.4.2. Reaction Force Constant Decomposition 

With respect to the reactive force constant, Figure 6 confirms and even emphasizes the results reported in Section 2.3 based on the Wiberg bond order analysis. Similarly to $\omega =\kappa_{\max}$, the position of the global maxima of $\kappa_{A1,A6}$ and $\kappa_{A4,A5}$ should indicate when each of the C-C bonds is formed. Based on that assumption, one can infer some insights from Figure 6. First, as the DA reaction becomes asynchronous, the $\kappa_{A1,A6}$ maximum shifts to the left, and its magnitude decreases, while the $\kappa_{A4,A5}$ maximum tends to fit $\kappa_{\max}$. Meanwhile, the global minimum of $\kappa_{A1,A6}$ tends to coincide with $\kappa_{\min}$. The typical inflection point and local maximum on the κ profile in the TS region of (moderate) asynchronous processes correspond to the global maximum of $\kappa_{A1,A6}$ and indicate, therefore, the formation of the fast-forming bond. This finding is in line with the analysis of bond orders reported above, which suggested that the first bond is increasingly formed around the TS when the reaction becomes asynchronous. Moreover, the position of $\kappa_{\min}$ with respect to the classical TS changes with the synchronicity of DA reactions. For synchronous reactions, $\kappa_{\min}$ is right-deviated as compared to the TS. Similar deviations have been found in other reactions, such as were reported in the reaction between HF and CO, where $\kappa_{\min}$ was located at the left of TS (ξ=−0.22) [29]. This deviation was found to be beyond any kind of statistical error of the method and may suggest an effect of the nuclear interaction on $\kappa_{\xi }$. 

It must be emphasized that the magnitude of the deviation between $\kappa_{\min}$ and TS decreases as the reaction becomes asynchronous. More interestingly, for likely two-step DA reactions (S > 0. 95Å), $\kappa_{\min}\;$ may be found at the left of the classical TS, such as in the case of **R994** (S = 1.07Å), where it is found at ξ=−0.23. For example, this gap is valued to ξ=0.90 in **R1310,** ξ=0.682 in **R218,**  ξ=0.45 in **R585,** ξ=0.23 in **R732**, and ξ=0.00 in **R173,** whose synchronicity indexes are 0.02, 0.24, 0.43, 0.67 and 0.84 Å, respectively. Therefore, the relative position of $\kappa_{\min}$ with respect to the TS seems to be an indicator of the degree of synchronicity in DA reactions, and in contrast with the profile of κ, the deviation between $\kappa_{\min}$ and the TS can be quantified. As such, it becomes possible to compare two reactions from the same synchronicity category, something which is not possible only through observation of the reaction force constant κ profile.

### 2.5. On the Validity and Limitations of Our Conclusions

This study has been carried out in the gas phase, as has been the case for many other investigations into the mechanism of the Diels–Alder reaction. However, it is not a secret to anybody that, in real situations, DA reactions are generally conducted in solvents or in the presence of catalysts [49]. Unfortunately, the computational cost associated with the explicit or even implicit inclusion of solvent effects, especially for the construction of IRC paths, was not affordable for such a large dataset. This justifies the choice made during this large-scale study. On the other hand, it is not easy to design an automated protocol for large-scale studies that can account for Lewis acid catalytic effects due to the specificity of each catalyst. Still, we do believe that these aspects are worth our efforts and should be addressed in our next investigations, certainly on a much-reduced dataset. Furthermore, we must admit that some of the quantitative mechanistic insights reported here, such as the boundaries of DA reactions classes in terms of their synchronicities, would change to the extent that still has to be appreciated if one considers the non-IRC effect on the reactions considered [50] or analyses the same problem from another view, such as the entropic change over the reaction path [51]. 

We would like to insist on the fact that this work was first intended to provide qualitative insights into the (a)synchronicity of DA reactions, keeping in mind that more accurate data in terms of activation energies, reaction force and reaction force constants would be generated at higher computational levels. Despite all of this, we have tried to assess the robustness of our qualitative conclusions in terms of basis set variability. For this purpose, we considered a hand-selected set of four DA systems and the much larger 6-31+G(d,p) basis set compared to the golden standard of DA reaction studies, 6-31G(d). Chosen systems included the DA reaction between 1,3-butadiene and ethylene (Sys1), cyanoethylene (Sys2), 1,1-dicyanoethylene (Sys3), as well as 1-methyl-1,3-butadiene and 1,1-dicyanoethyelene (Sys4). These systems were selected to cover a wide range of synchronicity indices. The quantitative parameters of interest, as well as RFA plots and atomic decomposition curves, are provided in Table S1 and files RFA and ARED, respectively. 

From Table S1, it can be seen that synchronicity indices are not much affected. Indeed, these were valued to 0.00, 0.48, 0.88 and 1.01Å at the B3LYP/6-31G(d) level, and 0.00, 0.50, 0.86 and 0.96Å using B3LYP/6-31+G(d,p) for Sys1, Sys2, Sys3 and Sys4, respectively. Note that the deviations between these series of values are lower than 0.05 Å. In terms of TS polarity, we noted changes of up to 0.016 e, representing less than 6%. These findings suggest an acceptable conservation of structural and electronic features when going from the 6-31G(d) to the 6-31+G(d,p) basis set. In addition, the two components of the activation energy, i.e., E_act1_ and E_act2_, were also computed. Our calculations suggest that E_act1_ ranges between 68.18 and 74.86% at the B3LYP/6-31G(d) level and from 71.25 to 76.02% at the B3LYP/6-31+G(d,p) level. These ranges are consistent with the range of 70–75% suggested in Section 2.2. More interestingly, figures in file RFA reveal that the shapes of E, F and κ are not affected by the choice of the basis set. For instance, regarding the graph of κ, one should notice a unique minimum, a minimum and a shoulder, two minima and a roughly zero maximum, and two minima alongside a positive maximum in the TS region of Sys1, Sys2, Sys3 and Sys4, respectively, and all of this is irrespective of the basis set used. Similarly, looking at the atomic resolution of energy derivatives for the same systems using the two basis sets, we noticed that all the qualitative features discussed in Section 2.4 are conserved. 

## 3. Methodology

### 3.1. Theoretical Background

#### 3.1.1. Reaction Force Analysis

The conversion of reactants into products during chemical reactions induces equivalent changes in the overall energy of the system [52]. As the reaction proceeds, atoms are displaced towards or away from each other, and their motions are associated with a force acting on the whole system to bring it from the initial to the final state. Torro-Labbé and coworkers showed that this force F (reaction force) could be defined as the opposite of the system’s potential energy E(ξ) with respect to the reaction coordinate ξ (Equation (1)) [53]. Most often, the reaction path ξ is chosen along the intrinsic reaction coordinate (IRC) path, which is the mass-weighted steepest-descent path connecting the reactants to the products through the TS [54].

(1) $$F\left(\xi \right)=-\frac{\mathrm{dE}\left(\xi \right)}{d\xi }$$

For a chemical process presenting a unique energy barrier, the profile of F splits the IRC path into three regions delimited by its global minimum α and maximum γ located at both sides of the TS [55]. The region between these extrema is known as the TS region and lies between the preparation phase, on its left, and the relaxation phase, situated on its right [56]. Previous studies have shown that the most important electronic changes take place in the TS region, while the other two regions are dominated by structural changes [43]. The fragmentation of the IRC path suggested by the profile of the reaction force allows for the partitioning of the activation energy in two terms, E_act,1_ and E_act,2_, defined in Equations (2) and (3). The first term (Equation (1)) accounts for the energy needed by the reacting system to overcome the structural resistance in the preparation phase, while Equation (3) evaluates the energy consumed from α to the TS.

(2) $$E_{\mathrm{act},1}=-\int_{R}^{\alpha }F\left(\xi \right)d\xi \;$$

(3) $$E_{\mathrm{act},2}=-\int_{\alpha }^{\mathrm{TS}}F\left(\xi \right)d\xi$$

In the same framework, Yepes et al. introduced the second derivative of E(ξ), known as the reaction force constant, and demonstrated its relevance in the investigation of reaction mechanisms [36].

(4) $$\kappa \left(\xi \right)=\frac{d^{2}E\left(\xi \right)}{d\xi^{2}}$$

The reaction force theory is one of the most powerful reactivity paradigms developed during the last two decades. This theory has been successfully applied to several types of reactions, such as the deamination of cytosine molecules [57] or intramolecular proton transfer reactions [58]. In contrast with the transition state theory [59], which considers a fixed position on the IRC path as the (classical) transition state, the reaction force theory defines a whole region, including the classical TS, where the most electronic changes of the reaction take place. The concept of the TS region is consistent with the TS spectroscopy of Zewai and Polanyi [60], which describes the TS region as a continuum of transient and unstable intermediate configurations of the reacting system.

#### 3.1.2. Atomic Resolution of Energy Derivatives

Although much can be learned from the profile of E, F, and κ as defined in Equations (1) and (4) [61], the chemistry behind a reaction happens between the interacting atoms. Therefore, resolving the reaction force F and reaction force constants κ in terms of atomic contributions should provide interesting information about the implication of atoms in various reaction stages.

In 2016, Jędrzejewski et al. [29] reported the mathematical foundations for the atomic resolution of energy derivatives along the IRC path. These authors showed that the reaction force F and force constant κ can be decomposed into atomic components by introducing the Hellman–Feynman [62] (H–F) forces acting on each nucleus in the standard definition of F and κ. Equation (5) shows that the contribution of an atom A to the reaction force F, noted $F_{A}\left(\xi \right)$, is the scalar product of the H–F force vector $F_{A}$ and the deviation vector $\frac{\partial R_{A}}{\partial \xi }$, denoting the change in the position of a given atom during the reaction.

(5) $$F_{\xi }=-\frac{\mathrm{dE}}{d\xi }=-\underset{A\in M}{\sum }\frac{\partial E}{\partial R_{A}}\;\frac{\partial R_{A}}{\partial \xi }=\underset{A\in M}{\sum }F_{A}\frac{\partial R_{A}}{\partial \xi }=\underset{A}{\sum }F_{A}\left(\xi \right)\;$$

(6) $$\kappa_{\xi }=-\frac{{\mathrm{dF}}_{\xi }}{d\xi }=-\underset{A\in M}{\sum }\frac{d}{d\xi }\left[F_{A}\frac{{\mathrm{dR}}_{A}}{d\xi }\right]=\underset{A}{\sum }\kappa_{A}\left(\xi \right)$$

where the sums run over all the atoms in the molecule. 

The foundations of this method rest on the work by Ordon and Komorowski [29], who investigated the energy derivatives within the conceptual density functional theory (DFT) framework [63] and the vibrational softening of molecules [64].

### 3.2. Computational Details

The main findings reported in this study were obtained using the AMADAR (Automated Workflow for Mechanistic Analysis of Diels–Alder Reaction) package recently developed in our research group [31] and freely accessible from GitHub (CMCDD/AMADAR, accessed on 14 October 2021). The package integrates several python-written modules working in a consortium to generate (an unlimited number of) DA transition state geometries and perform subsequent IRC-based analyses. The AMADAR program only needs to be fed with the SMILES strings of the cycloadducts, and for usage, requires both the RDKit toolkit [65] and an electronic structure software (Gaussian 09 [66] in this case) to be already installed on the computing platform. Two special modules of the package perform, from IRC paths, the reaction force analyses and atomic decomposition of the reaction force and reaction force constant. 

In this study, SMILES strings of 2000 likely Diels–Alder cycloadducts were retrieved from the ZINC database [67] (SMILES provided in the Supplementary Materials) and pumped into the AMADAR pipeline. These compounds were randomly sampled from an original dataset of 5.7 million compounds containing at least one cyclohexene substructure. ZINC is a structural bank of commercially available chemicals collected from diverse catalogs of different vendors [67]. Using the default settings of the AMADAR program, each SMILES string was embedded into a 3D box and minimum-optimized using the universal force-field (UFF) developed in Rappé’s group [68]. Then, the returned conformer was subjected to a constrained optimization in internal coordinates, coercing the system to adopt a new configuration in which the terminal C atoms of diene and dienophile moieties were placed 2.15 Å apart (pseudo-guess TS). This two-fragments structure was then sequentially refined using the single-ended Berny algorithm [69] at the semi-empirical PM6 [70] and quantum mechanics B3LYP/6-31G(d) [71,72] levels. For each TS predicted, natural bond order (NBO) [73] calculations were performed to evaluate the Wiberg bond orders of the two emerging C-C bonds as well as the charge transfer between the diene and dienophile in the TS geometry. The latter was identified with the absolute value of the global NPA charge on each fragment. It is worth noting that the B3LYP/6-31G(d) level of theory has been the standard level of calculations in previous investigations on the mechanism of the Diels–Alder reaction, as it offers a good compromise between accuracy and computational cost for simple DA reactions [23,32,74]. Furthermore, in a benchmark study published in 2001 on the Diels–Alder reaction of butadiene and cyclic five-membered dienes with ethylene, it was found that the B3LYP functional gives results in good agreement with CCSD(T)/6-31G(d) [75]. More interestingly, the changes as a function of the basis set were very small, so that B3LYP/6-31G(d) can be considered an adequate level to accurately model Diels–Alder reactions.

Furthermore, 150 of the predicted TSs were used for determining the IRC paths with a step size of 0.8 (amu)^1/2^Bohr at the B3LYP/6-31G(d) level. The choice of these DA systems was driven by the need to cover a wide range of synchronicity indexes, measured as the difference of length between the two emerging C-C bonds at the TS. The IRC paths constructed were first examined in the framework of the reaction force theory. Then, the reaction force and reaction force constants were decomposed in terms of atomic (fragment) contributions using the LOK formalism [29] implemented in the AMADAR package. Finally, to assess the bond breaking/formation processes during the reaction, NBO calculations were performed at the B3LYP/6-31G(d) level on the geometries extracted from the IRC paths.

## 4. Conclusions

In this paper, the perpetual question of (a)synchronicity in Diels–Alder (DA) reactions has been investigated using the reaction force theory and the atomic resolution of energy derivatives. The findings suggested that the transition between preferentially concerted to stepwise DA processes can be cautiously located in the range of synchronicity indexes from 0.90 to 1.00 Å. Furthermore, the global maximum of the reaction force constant seems to indicate the formation of a slow-forming C-C bond. In addition, the atomic resolution of energy derivatives suggested that the mechanism of the DA reaction involves two inner elementary processes associated with the formation of the C-C bonds. The position of the global minimum of the reaction force constant with respect to the TS is shown to correlate with the synchronicity of DA reactions. Further studies are being conducted to understand common features in the reaction electron flux along the IRC path of this huge dataset of DA reactions. Further investigations are in preparation in our lab to perform more systematic statistical analyses of the data collected.

## Figures and Tables

**Table 1:** Activation energy decomposition and its relationship with the polarity and synchronicity for a representative set of twenty reaction features. E_act_, E_act,1_ and E_act,2_ expressed in kcal/mol.

Reaction ID	GEDT(e)	S (Å)	E_act_	E_act,1_	E_act,2_
R76	0.000	0.00	20.58	15.73	4.48
R283	0.032	0.09	23.04	17.83	5.21
R313	0.002	0.12	20.70	15.79	4.91
R525	0.031	0.18	23.97	19.49	4.48
R1336	0.051	0.25	16.64	12.30	4.34
R568	0.011	0.27	22.79	18.03	4.76
R359	0.107	0.37	21.69	16.56	5.13
R725	0.112	0.38	20.74	15.62	5.12
R1669	0.122	0.43	20.55	15.52	5.03
R279	0.084	0.44	17.09	12.65	4.44
R793	0.081	0.54	19.06	14.64	4.42
R318	0.113	0.60	19.04	14.60	4.44
R972	0.180	0.65	20.06	15.23	4.83
R1453	0.089	0.76	23.73	18.87	4.86
R1048	0.094	0.77	24.50	17.95	6.56
R1439	0.128	0.80	19.02	14.28	4.74
R727	0.153	0.82	21.38	16.48	4.10
R407	0.138	0.95	19.65	14.80	4.85
R1018	0.211	0.97	23.04	16.21	6.82
R994	0.247	1.07	19.57	14.29	5.28

## Data Availability

All the data supporting the findings reported in this study can be obtained either by request to the corresponding author or in the supplementary materials.

## Supplementary Materials

The following are available online, SMILES_cycloadducts_and_reactants.csv, TS.txt (optimized geometries of over 1900 DA transition states), Rx_numbering.txt (correspondence between the automatically generated TS filenames and the numbering used in this study), Figure S1: Correlation between activation energy and polarity (GEDT), Figure S2: Bond order and atomic charge analysis of a complementary set of five Diels–Alder reactions, Table S1: On the variability in quantitative data due to basis set change, RFA: reaction force analysis of Sys1, Sys2, Sys3 and Sys4 at the B3LYP/6-31g(d) and B3LYP/6-31+G(d,p) levels, ARED: atomic resolution of energy derivatives for Sys1, Sys2, Sys3 and Sys4 at the B3LYP/6-31g(d) and B3LYP/6-31+G(d,p) levels.
