# Supplementary material for: New Insights into the (A)Synchronicity of Diels–Alder Reactions: A Theoretical Study Based on the Reaction Force Analysis and Atomic Resolution of Energy Derivatives
Source: Molecules. 2022 Feb 25;27(5):1546. doi: 10.3390/molecules27051546 (PMC8911883; doi:10.3390/molecules27051546)
Supplement: Supplementary file 1 [file molecules-27-01546-s001.zip › molecules-1572309-Table S3.pdf]

Table S1. Basis set effect on energetic and structural parameters of a selected set of four DA reactions. Results obtained using the 6-31+G(d,p) basis set are given

Reaction ID	GEDT(e)	S (Å)	E_{act}	$E_{act,1}$	$E_{act,2}$	%E_{act1}	%E_{act2}
			**6-31G(d)**				
**Sys1**	0.000	0.00	19.29	14.44	4.85	74.86	25.14
**Sys2**	0.116	0.48	16.76	12.21	4.55	72.85	27.15
**Sys3**	0.243	0.88	12.57	8.57	3.83	68.18	31.82
**Sys4**	0.277	1.01	10.25	7.55	2.7	73.64	26.36
			**6-31+G(d,p)**				
**Sys1**	0.000	0.00	21.06	16.01	5.05	76.02	23.98
**Sys2**	0.122	0.50	18.38	13.66	4.72	74.31	25.69
**Sys3**	0.256	0.86	13.74	9.79	3.95	71.25	28.75
**Sys4**	0.293	0.96	11.43	8.5	2.94	74.37	25.63
