# Supplementary figures and images for: New Insights into the (A)Synchronicity of Diels–Alder Reactions: A Theoretical Study Based on the Reaction Force Analysis and Atomic Resolution of Energy Derivatives
Source: Molecules. 2022 Feb 25;27(5):1546. doi: 10.3390/molecules27051546 (PMC8911883; doi:10.3390/molecules27051546)

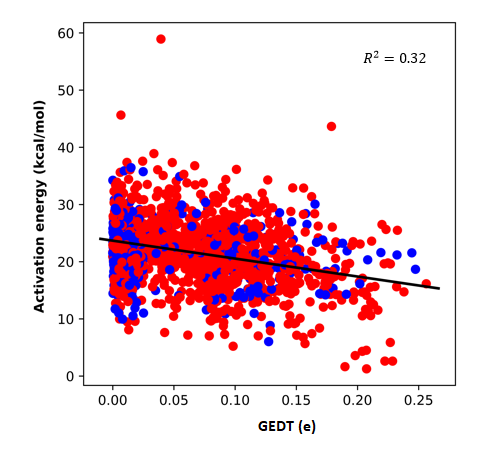

Supplement: Supplementary file 1 [file molecules-27-01546-s001.zip › Figure S1.png]

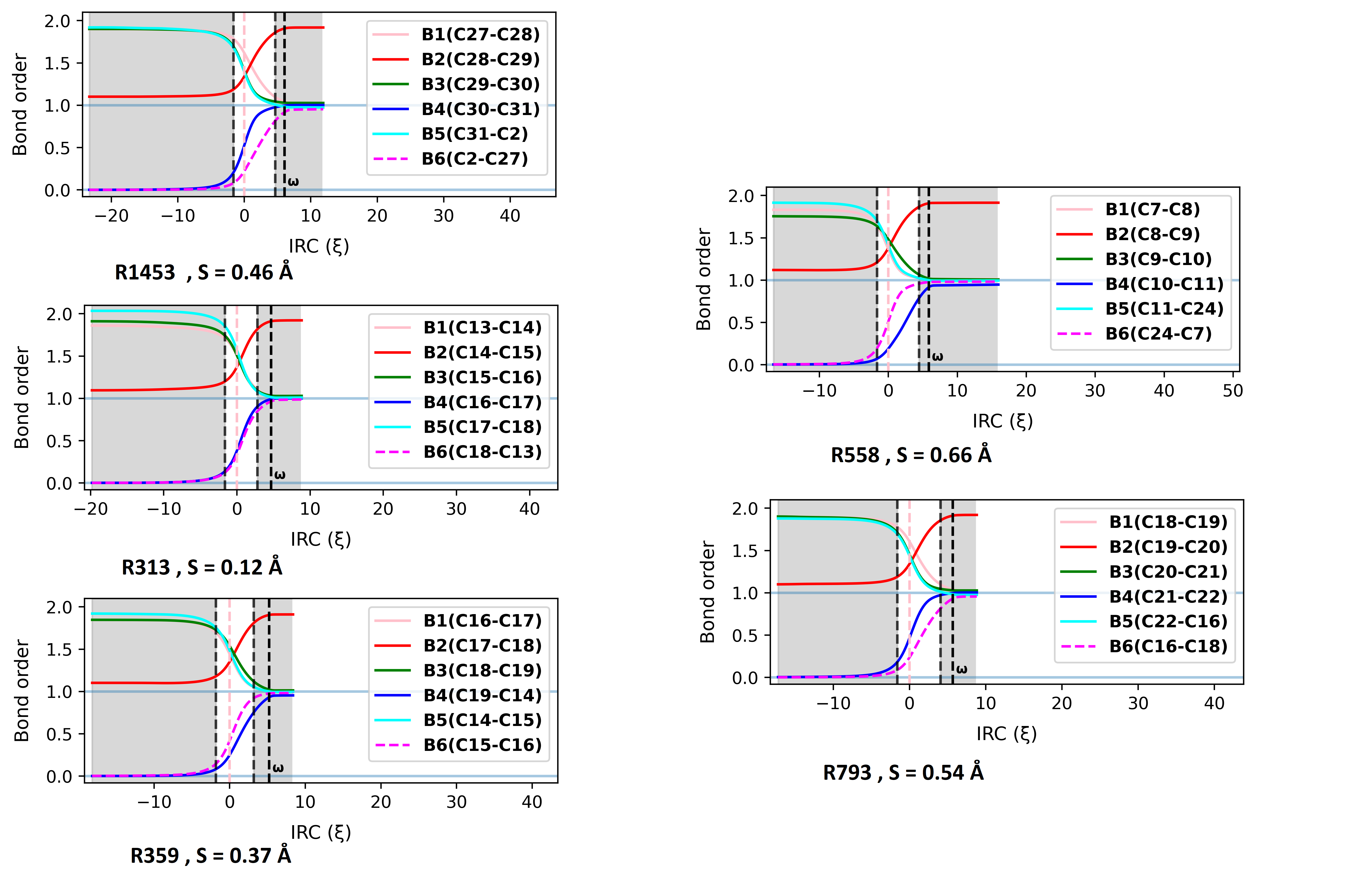

Supplement: Supplementary file 1 [file molecules-27-01546-s001.zip › Figure S2.png]
